# Highest Recorded Serum Creatinine

**DOI:** 10.1155/2021/6048919

**Published:** 2021-10-14

**Authors:** Christine Persaud, Uttsav Sandesara, Victor Hoang, Joshua Tate, Wayne Latack, David Dado

**Affiliations:** ^1^Department of Medicine, Keesler Medical Center, Biloxi 39534, MS, USA; ^2^Department of Medicine, University of Mississippi Medical Center, Jackson 39216, MS, USA

## Abstract

Serum creatinine is a commonly used laboratory marker to assess kidney function; however, there has not been an established level of serum creatinine to predict mortality. After extensive literature review, we present a case of the highest recorded serum creatinine of 73.8 mg/dL in a 23-year-old male with the history of pediatric deceased donor kidney transplant (DDKT). He initially presented with uremia and signs of acute renal allograft failure after two months of immunosuppressive medication nonadherence, ultimately requiring emergent hemodialysis, which was complicated by new onset seizures. This was the patient's fourth episode of late acute rejection and emphasizes the need for education of immunosuppressant adherence and periodic monitoring of renal function in high-risk patients. Though there is no known creatinine level incompatible with life, this patient appears to have the highest known serum creatinine in a uremic patient on record.

## 1. Introduction

Serum creatinine is a commonly used laboratory measurement to gauge renal function in clinical practice. Creatinine is a byproduct of the metabolism of creatine in skeletal muscle and dietary meat intake [1]. It is freely filtered across the glomerulus, and there is additional creatinine secretion in the proximal tubule of the nephron via organic cation secretory pathways [2]. Therefore, serum creatinine is a reflection of glomerular filtration rate, creatinine secretion in the renal tubule, creatine intake, and baseline skeletal muscle mass. Creatinine is also known to vary by gender and race, with average values of 1.14 mg/dL in men and 0.93 mg/dL in women in the United States [3]. The previously highest serum creatinine reported was 53 mg/dL [4]. In current clinical practice, there remains uncertainty regarding the role of serum creatinine and mortality, specifically in the context of the degree of creatinine elevation. We present a case of acute renal allograft rejection with the highest serum creatinine reported in previous literature.

## 2. Case Presentation

We report a 23-year-old African American male with a medical history of pediatric DDRT secondary to left-sided dysplastic kidney and right-sided obstructive uropathy. He presented to the emergency department with a one-week history of decreasing urine output, increased shortness of breath, generalized weakness, and nausea. He initially received his DDKT in 2010, which has been complicated by multiple episodes of both acute T cell-mediated and acute antibody-mediated rejection. The patient admitted to a two-month history of immunosuppressive medication nonadherence with tacrolimus, mycophenolate, and prednisone.

Physical examination revealed a young male, in no distress, weighing 142 pounds. He had a blood pressure of 153/102 mmHg and a heart rate of 77 beats/min. Exam findings are as follows: pale conjunctiva, lungs clear to auscultation, regular heart rate and rhythm, active bowel sounds, no abdominal tenderness, and no focal neurological deficit. Admission labs included a serum creatinine of 64.6 mg/dL (0.67–1.17 mg/dL) with a blood urea nitrogen (BUN) of 212 mg/dL (6–20 mg/dL), which increased to a serum creatinine of 73.8 mg/dL and BUN of 244 mg/dL prior to initiation of emergent dialysis. Serum chemistry included sodium 137 mmol/L (136–145 mmol/L), potassium 6.0 mmol/L (3.5–5.1 mmol/L), chloride 98 mmol/L (98–107 mmol/L), total carbon dioxide 7 mmol/L (22–29 mmol/L), glucose 68 mg/dL (74–106 mg/dL), and phosphorus 14.7 mg/dL (2.7–4.5 mg/dL).

The patient was initiated on emergent dialysis via a temporary dialysis catheter. Dialysis was complicated by new onset of seizures, initially thought to be secondary to dialysis disequilibrium syndrome. An electroencephalogram (EEG) was performed showing right temporal spikes epileptiform discharges with intermittent slow waves lateralized to the right hemisphere. Patient was thought to have epilepsy with initial seizure exacerbation secondary to severe metabolic derangements in setting of acute renal failure and severe azotemia. He was initiated on daily levetiracetam with resolution of seizures.

Patient received continuous renal replacement therapy (CRRT) while in the intensive care unit. After stabilization of his acute medical issues, a tunneled dialysis catheter was placed in his right internal jugular vein prior to discharge. Ultimately, the patient was diagnosed with renal allograft failure secondary to medication nonadherence and was discharged home on intermittent hemodialysis with improvement in serum creatinine to 23.8 mg/dL and BUN of 76 mg/dL at time of discharge, after completion of four hemodialysis sessions.

## 3. Discussion

This case demonstrated a record high serum creatinine of 73.8 mg/dL based on our review of previous literature [4–7]. Currently, there is not a known effect of creatinine levels on human physiology or survivability [5]. Even with its inherent limitations, previous studies have explored the relationship between increased serum creatinine and mortality. In a prospective population-based study of people >65 years old, 11.2% of the population had an elevated serum creatinine (>1.5 mg/dL in men and >1.3 mg/dL in women) which was associated with a higher overall mortality (76.7 vs. 29.5/1000 years) [8]. In a retrospective study of hospitalized patients, very low serum creatinine admission value of <0.4 mg/dL was significantly associated with increased mortality, exceeding the risk related to a creatinine value of >1.5 mg/dL [9].

This patient had elevated creatinine secondary to acute renal allograft rejection. Acute allograft rejection is a functional deterioration of the allograft caused by specific pathologic changes due to recipient's immune system recognizing nonself-antigens from the allograft [10]. Typically, such rejection leads to subsequent rise in serum creatinine indicating substantial histological damages without significant symptom manifestation [11]. The long-term function of a renal allograft is associated significantly with acute rejection episodes, especially late acute rejections [12]. Late acute rejections occur greater than 3 months after transplant, and this case represented the patient's fourth episode of such. In a previous historical cohort study, the late acute rejection group was shown to have the highest amount of graft loss when compared to the no rejection and early acute rejection groups [12]. This patient was ultimately diagnosed with renal allograft failure and remained dialysis dependent after discharge.

Our patient showed minimal symptoms initially, likely related more to azotemia and metabolic derangements rather than the elevated serum creatinine level. Chronic kidney disease (CKD) symptoms typically do not manifest until KDIGO CKD stages IV and V [13]. Chronic kidney disease is commonly detected by routine urine testing and labs, as it initially can present asymptomatically [13]. This case demonstrates the importance of periodic lab evaluation to assess kidney function in high-risk renal patients and the need for counseling on the importance of medication adherence. There is no known creatinine incompatible with life, and this patient appears to have the highest survivable serum creatinine recorded in the medical literature.
